# Disrupting ROS-protection mechanism allows hydrogen peroxide to accumulate and oxidize Sb(III) to Sb(V) in *Pseudomonas stutzeri* TS44

**DOI:** 10.1186/s12866-016-0902-5

**Published:** 2016-11-25

**Authors:** Dan Wang, Fengqiu Zhu, Qian Wang, Christopher Rensing, Peng Yu, Jing Gong, Gejiao Wang

**Affiliations:** 1 State Key Laboratory of Agricultural Microbiology, College of Life Science and Technology, Huazhong Agricultural University, Wuhan, People’s Republic of China; 2 College of Resources and Environment, Fujian Agriculture and Forestry University, Fuzhou, People’s Republic of China

**Keywords:** *Pseudomonas stutzeri*, Sb(III) oxidation, H_2_O_2_, Transposon mutagenesis, *gshA*, Reactive oxygen species (ROS)

## Abstract

**Background:**

Microbial antimonite [Sb(III)] oxidation converts toxic Sb(III) into less toxic antimonate [Sb(V)] and plays an important role in the biogeochemical Sb cycle. Currently, little is known about the mechanisms underlying bacterial Sb(III) resistance and oxidation.

**Results:**

In this study, Tn5 transposon mutagenesis was conducted in the Sb(III)-oxidizing strain *Pseudomonas stutzeri* TS44 to isolate the genes responsible for Sb(III) resistance and oxidation. An insertion mutation into *gshA,* encoding a glutamate cysteine ligase involved in glutathione biosynthesis, generated a strain called *P. stutzeri* TS44-gshA_540_. This mutant strain was complemented with a plasmid carrying *gshA* to generate strain *P. stutzeri* TS44-gshA-C. The transcription of *gshA*, the two superoxide dismutase (SOD)-encoding genes *sodB* and *sodC* as well as the catalase-encoding gene *katE* was monitored because *gshA*-encoded glutamate cysteine ligase is responsible for the biosynthesis of glutathione (GSH) and involved in the cellular stress defense system as are superoxide dismutase and catalase responsible for the conversion of ROS. In addition, the cellular content of total ROS and in particular H_2_O_2_ was analyzed. Compared to the wild type *P. stutzeri* TS44 and TS44-gshA-C, the mutant *P. stutzeri* TS44-gshA_540_ had a lower GSH content and exhibited an increased content of total ROS and H_2_O_2_ and increased the Sb(III) oxidation rate. Furthermore, the transcription of *sodB*, *sodC* and *katE* was induced by Sb(III). A positive linear correlation was found between the Sb(III) oxidation rate and the H_2_O_2_ content (*R* ^2^ = 0.97), indicating that the accumulated H_2_O_2_ is correlated to the increased Sb(III) oxidation rate.

**Conclusions:**

Based on the results, we propose that a disruption of the pathway involved in ROS-protection allowed H_2_O_2_ to accumulate. In addition to the previously reported enzyme mediated Sb(III) oxidation, the mechanism of bacterial oxidation of Sb(III) to Sb(V) includes a non-enzymatic mediated step using H_2_O_2_ as the oxidant.

**Electronic supplementary material:**

The online version of this article (doi:10.1186/s12866-016-0902-5) contains supplementary material, which is available to authorized users.

## Background

Antimony (Sb) is a metal belonging to group V in the periodic table. It is present in aquatic systems and soil, with stibnite (Sb_2_S_3_) representing the most common mineral form [1]. Antimonials have been used to treat leishmaniasis for over 60 years [2, 3]. However, the U.S. Environmental Protection Agency recognized Sb as a priority pollutant [4], and the WHO has proposed 5 μg/L to be the highest acceptable Sb concentration in potable water [5]. Although Sb pollution has gained increased attention in recent decades [6–8], the exact mechanism of Sb toxicity in both mammals and microorganism remains unclear [9, 10].

A comparison of two common inorganic forms of Sb revealed that antimonite [Sb(III)] was more toxic than antimonate [Sb(V)] [7]. The abiotic Sb(III) oxidation in the natural environment is extremely slow [11]. In contrast, Sb(III)-oxidizing bacteria can oxidize Sb(III) at a relatively high rate. Thus, Sb(III) oxidation was proposed to be a microbial detoxification process that could be useful for environmental Sb bioremediation [12–14]. A few Sb(III)-oxidizing bacteria had been reported in earlier literature [15, 16]. Recently, dozens of Sb(III)-oxidizing bacteria have been isolated and identified both by our group and by others [12, 13, 17, 18]. The arsenite oxidase AioAB in *Agrobacterium tumefaciens* was also found to be able to oxidize Sb(III) [19]. In addition, the oxidoreductase AnoA was shown to be responsible for bacterial Sb(III) oxidation [20]. However, the disruption of both of these genes did not result in a complete loss of Sb oxidation, indicating the existence of other mechanisms responsible for bacterial Sb(III) oxidation.

In general, aerobic respiration is inevitably accompanied by the production of reactive oxygen species (ROS). ROS include superoxide (O_2_^•-^), hydroxyl (OH^•^), hydroperoxyl (HO_2_^•^), and peroxyl (RO_2_^•^) radicals and other oxidizing agents, such as hydrogen peroxide (H_2_O_2_) [21]. Among the various ROS, O_2_^•-^ is the primary radical that results from the univalent reduction of molecular oxygen (O_2_) via the bacterial respiratory chain. Subsequently, O_2_^•-^ can be converted into H_2_O_2_ and O_2_ by superoxide dismutase (SOD). Both O_2_^•-^ and H_2_O_2_ can generate highly reactive hydroxyl radicals via the Haber-Weiss reaction or the Fenton reaction [22]. ROS can damage different types of macromolecules; thus, bacteria have evolved defense mechanisms against ROS and induce specific genes in response to oxidative stress [23]. Glutathione (GSH) is the central substrate involved in cellular protection against ROS and their toxic products in eukaryotic cells [24]. In bacteria GSH plays multiple role in protection against environmental stresses such as osmotic shock and acidity, as well as against toxins and oxidative stress. Indeed, GSH represents the primary low molecular weight thiol in many Gram-negative bacteria [25]. Two enzymes (glutamate cysteine ligase, the rate-limiting enzyme, and GSH synthetase) catalyze the *de novo* synthesis of GSH. These two enzymes are encoded by distinct genes (*gshA* and *gshB*, respectively) in most bacteria [26].

Previously, the aerobic, arsenite-oxidizing bacterium *Pseudomonas stutzeri* TS44 was isolated from arsenic-contaminated soil [27] and the genome sequence was published (Accession No. AJXE00000000, [28]). In this study, we showed that strain TS44 is able to oxidize Sb(III) to Sb(V). Therefore, in an effort to determine the molecular mechanism underlying bacterial Sb(III) oxidation, more than 3000 Tn5 transposon insertion mutants were generated and screened. We isolated a mutant of *gshA* (TS44-gshA_540_) that showed an increase in Sb(III) oxidation rate. The Sb(III) oxidation rates of the wild type, mutant and complemented strain, the gene transcription of *gshA*, *sod* and *katE*, as well as the cellular contents of ROS, GSH and H_2_O_2_ were analyzed since *gshA* is responsible for the biosynthesis of GSH and therefore involved in the cellular stress defense system.

## Methods

### Bacterial strains, plasmids, primers and culture conditions

The strains, plasmids and primers used in this study are listed in Table 1 and Additional file 1: Table S1. The *Pseudomonas stutzeri* strains were grown in a chemically defined medium (CDM) [29] containing 0.114 mmol/L phosphate (0.07 mmol/L K_2_HPO_4_•3H_2_O and 0.044 mmol/L KH_2_PO_4_) and shaken under aerobic conditions at 28 °C. The *Escherichia coli* strains were cultured at 37 °C in Luria-Bertani medium [30]. Rifampin (Rif, 50 mg/mL), kanamycin (Kan, 50 mg/mL), tetracycline (Tet, 5 mg/mL) or chloromycetin (Cm, 50 mg/mL) were added when needed.

**Table 1:** The strains and plasmids used in this study

Strain/plasmid	Relevant properties or derivation	Source or reference
*Pseudomonas stutzeri*		
TS44	Wild-type, As(III)-oxidizing phenotype	[27]
TS44-gshA_540_	Kan^r^; *gshA* mutant by Tn5 random transposon mutagenesis	This study
TS44-gshA-C	Kan^r^ Cm^r^; *gshA* complemented strain	This study
TS44 (P*gshA*)	Kan^r^; TS44 with pLSP-P*gshA*	This study
TS44 (P*sodB*)	Kan^r^; TS44 with pLSP-P*sodB*	This study
TS44 (P*sodC*)	Kan^r^; TS44 with pLSP-P*sodC*	This study
TS44-gshA_540_ (P*gshA*)	Kan^r^; TS44-gshA_540_ with pLSP-P*gshA*	This study
TS44-gshA_540_ (P*sodB*)	Kan^r^; TS44-gshA_540_ with pLSP-P*sodB*	This study
TS44-gshA_540_ (P*sodC*)	Kan^r^; TS44-gshA_540_ with pLSP-P*sodC*	This study
*Escherichia coli*		
DH5α	supE44 lacU169(j80lacZM15) hRDR17 recA1 endA1 gyrA96 thi-1 relA1	[35]
S17-1	Pro^−^ Mob^+^; conjugation donor	[36]
pir116	mcrA, Δ(mrr-hsdRMS-mcrBC) recA1 R6Kγ lacZΔM15 λpir	Epicenter, Madison, WI
Plasmids		
pRL27-Kan	Kan^r^ Transposon vector, *ori*R6K	[37]
pCT-Zori	broad host vector, pUC ori, Cm^r^	[34]
pCT-Zori-*gshA*	gshA complementation vector	This study
pLSP-kt2lacZ	Kan^r^ *oriV*; *lacZ* fusion vector used for *lacZ* fusion constructs	T. R. McDermott, MSU
pLSP-P*gshA*	pLSP-kt2lacZ containing *gshA* promoter region	This study
pLSP-P*sodB*	pLSP-kt2lacZ containing *sodB* promoter region	This study
pLSP-P*sodC*	pLSP-kt2lacZ containing *sodC* promoter region	This study

### Isolation of a Sb(III) sensitive transposon mutant and complementation of the mutant strain

Plasmid pRL27-Kan was transferred into *P. stutzeri* TS44 by conjugation from *E. coli* strain S17-1 carrying Tn5 as described previously [31]. To obtain Sb(III) sensitive mutants, the colonies of transformants from the mating plates were spread onto LB agar plates containing 50 μg/mL Kan and 50 μg/mL Rif, in the presence or absence of 0.4 mmol/L C_8_H_4_K_2_O_12_Sb_2_S_3_(H_2_O) [as Sb(III)]. The mutants did not grow in the presence of the indicated concentration of Sb(III). The insertion sequence of the mutant was determined using a plasmid rescue strategy as described by Zheng et al. [31]. The sequences were compared to the draft genome sequence of *P. stutzeri* TS44 using BLAST [32] and compared to the protein sequence database at GenBank using the BlastX algorithm [33]. Finally, the Sb-sensitive Tn5-insertion mutant TS44-gshA_540_ was isolated.

The construction of the *gshA* complementary strain TS44-gshA-C was accomplished using the high-copy broad host range plasmid pCT-Zori containing a Kan resistance determinant ([34], Table 1). Briefly, a 2023 bp DNA fragment containing the complete *gshA* coding region along with 156 bp upstream and 289 bp downstream sequence was PCR-amplified using the gshA-F/gshA-R primers (Additional file 1: Table S1) and subsequently cloned into *Hin*dIII + *Bam*HI-digested pCT-Zori. The resulting plasmid pCT-Zori-*gshA* was transformed into *E. coli* S17-1 and conjugated into the mutant TS44-gshA_540_, yielding the complementary strain TS44-gshA-C. The integrity of the mutant and the complementary strain was confirmed by PCR amplification and subsequent DNA sequencing.

### Growth and Sb(III) oxidation tests

Overnight cultures of *P. stutzeri* strains TS44, TS44-gshA_540_ and TS44-gshA-C (OD_600_ = 1.0) were inoculated into 100 mL of CDM medium with or without 0.2 mmol/L Sb(III) and incubated at 28 °C for 48 h with shaking at 170 rpm. Culture samples were collected to determine the OD_600_ value using a UV spectrophotometer (DU800, Beckman, USA) at the designated time points. Sb(III)/Sb(V) concentrations were monitored using hydride-generation atomic fluorescence spectroscopy combining HPLC (HPLC-HG-AFS, Beijing Titan Instruments Co., Ltd., China) according to the method described by Li et al. [13]. The measured data were analyzed with single factor analysis of variance (one way ANOVA) method in Excel program.

### GshA activity and determination of GSH content

To detect GshA activity and GSH content, *P. stutzeri* strains TS44, TS44-gshA_540_ and TS44-gshA-C were cultured under aerobic condition with shaking in 100 mL of liquid CDM medium at 28 °C. Sb(III) was added at a final concentration of 0.2 mmol/L when the OD_600_ reached 0.3. Cultures without the addition of Sb(III) were used as controls. One mililiter of cells was centrifuged at 13,400 × g at 4 °C after 30 min of cultivation. The pellet was washed twice using phosphate-buffered saline (PBS, pH 7.0), then resuspended in 1 mL of PBS and sonicated on ice to dissolve the cell membranes. The total protein content of the sonicate was measured by the Coomassie brilliant blue G-250-staining method [38]. Bovine serum albumin was used as the standard.

The GshA activity and GSH content were determined using 2, 3-naphthalenedicarboxyaldehyde (NDA) (Aladdin Industrial Co., Shanghai, China) as described previously [39]. To measure GshA activity, an equal volume of cell lysate was mixed with the GshA reaction buffer and substrate solution (50 μL each). Fifty microliter of reaction terminator (1 mol/L Na_2_CO_3_) was added after 45 min of incubation at 28 °C. The mixture was incubated on ice for 20 min and centrifuged at 8000 × g for 5 min. Then, 20 μL of the supernatants were transferred to wells of black Microlon 96-well plates (Greiner). The samples were mixed with 180 μL of NDA derivatization solution (50 mmol/L Tris–HCl, pH = 10, 0.5 N NaOH, and 10 mmol/L NDA in Me_2_SO, v/v/v 1.4/0.2/0.2). Fluorescence was measured (472 ex/528 em) with an EnVision® Multimode Plate Reader (Perkin Elmer) after 60 min of incubation at 37 °C and converted to the γ-glutamylcysteine concentration using appropriate calibration standards. The procedure for measuring GSH content was almost the same as the procedure to detect GshA activity, except that the reaction terminator was added immediately when the equal volume of cell lysate was mixed with the GshA reaction buffer and substrate solution (50 μL each).

### Determination of the ROS content

The cellular ROS content was tested with the fluorescent probe 2, 7-dichlorofluorescin diacetate (DCFH-DA) (Sigma Chemical Co., St. Louis, MO, USA). The cells were cultivated and collected for processing as described above. Cells from 0.5 mL cultures were washed two times with PBS buffer and resuspended in 0.5 mL of PBS buffer. Then, a 0.2 mL cell suspension was mixed with 5 μL of 0.1 mmol/L DCFH-DA and incubated at 37 °C for 30 min to develop the fluorescent product DCF. The cells were harvested by centrifugation for 3 min at 13,400 × g and washed two times using PBS buffer (pH 7.0) to remove background fluorescence. The fluorescence was measured (488 ex/535 em) on the EnVision® Multimode Plate Reader (Perkin Elmer) [40]. The ROS contents were normalized to the total protein content as described above.

### Quantification of gene expression using a *lacZ* reporter gene fusion and qRT-PCR

Quantitative reverse transcription PCR (qRT-PCR) was employed to test the transcription of *gshA*, two superoxide dismutase (SOD)-encoding genes (*sodB* and *sodC*) and the catalase *katE*. The strains were inoculated into 100 mL of CDM medium. 0.2 mmol/L of Sb(III) was added (or not) when the OD_600_ reached 0.3. The cells were harvested after 30 min. Total RNA was extracted using the TRIzol® Reagent (Invitrogen) and treated with DNaseI following the manufacturer’s instructions. The synthesis of cDNA from 300 ng of total RNA was performed using the RevertAid First Strand cDNA Synthesis Kit (Thermo) [41]. The resulting cDNA was used as a template for qRT-PCR with the SYBR-Green® qPCR Master Mix (Takara). Primers RT-gshA-F/RT-gshA-R, RT-sodB-F/RT-sodB-R, RT-sodC-F/RT-sodC-R and RT-katE-F/RT-katE-R were used to test the expression of *gshA*, *sodB*, *sodC* and *katE*, respectively. The RT-PCRs were performed using the AB ViiA 7 RT-PCR system (Life Technologies) following the manufacturer’s recommended protocol. The annealing temperature for *gshA* and *sodB* was 55 °C, while for *sodC* and *katE* it was 48 °C.

For the *lacZ* reporter fusion analysis, plasmid pLSP-kt2lacZ was used to construct the *lacZ* fusions. DNA fragments containing the predicted promoter regions of *gshA*, *sodB* and *sodC* were amplified by PCR using the primers PgshA-F/PgshA-R, PsodB-F/PsodB-R and PsodC-F/PsodC-R, respectively. The DNA fragments were digested using *Eco*RI and *Bam*HI and ligated into the double-digested pLSP-kt2lacz. The resulting plasmids pLSP-P*gshA*, pLSP-P*sodB*, and pLSP-P*sodC* were separately transferred into strains TS44 and TS44-gshA_540_ by conjugation employing *E. coli* S17-1. The resulting strains containing the above constructs were inoculated into 100 mL of CDM medium and cultivated with shaking at 28 °C. 0.2 mmol/L of Sb(III) was added when the OD_600_ reached 0.3. The samples were collected and the β-galactosidase activity was tested according to the method described by Miller [42] after 30 min of incubation.

### Determination of the linear correlation between H_2_O_2_ and Sb(III) oxidation rate

To test the H_2_O_2_ content, strains TS44, TS44-gshA540 and TS44-gshA-C were incubated as described above. To eliminate the H_2_O_2_ content difference caused by the different amount of cell collection, Sb(III) was added when the OD_600_ reached 0.3, and then 2 mL of cells were harvested after a 30 min incubation with Sb(III). The cells were resuspended in 1 mL of K_3_PO_4_ (pH 7.8) after washing twice with 50 mmol/L K_3_PO_4_ (pH 7.8) and sonicated on ice. Then, the sonicated cell lysates were centrifuged at 13,400 × g at 4 °C, and 0.1 mL of the supernatant was transferred to wells of black Microlon 96-well plates (Greiner). The samples were mixed with 50 μL of amplex red (AR) (Sigma Chemical Co., St. Louis, MO, USA) and 50 μL of horseradish peroxidase (HRP) (F. Hoffmann-La Roche Ltd, Shanghai, China), then incubated at 37 °C for 15 min. Fluorescence was measured (530 ex/587 em) as described above using the EnVision® Multimode Plate Reader (Perkin Elmer) and converted to the H_2_O_2_ concentration using appropriate calibration standards [43].

To detect the dynamic variation of the H_2_O_2_ and Sb(III) contents, strains TS44, TS44-gshA_540_ and TS44-gshA-C were inoculated into 100 mL of CDM medium supplemented with 0.2 mmol/L Sb(III). The cells were harvested and sonicated as described above at the designated time points. The Sb(III) contents were monitored using HPLC-HG-AFS as described above, while the H_2_O_2_ contents were determined by the AR/HRP method as described above. A boiled CDM culture of strain TS44-gshA_540_ was used as a control. To test the effect of H_2_O_2_ on bacterial growth, strain TS44 was inoculated onto CDM plates containing different concentrations of H_2_O_2_ and cultivated at 28 °C for 48 h.

In vitro oxidation of Sb(III) by H_2_O_2_ was performed using an un-inoculated liquid CDM medium containing 0.2 mmol/L of Sb(III) and the media with the addition of 0, 0.02, 0.05, 0.1, 0.3, 0.4 and 0.5 mmol/L of H_2_O_2_ and reacted within 10 min.

## Results

### A Sb(III)-sensitive mutant could be generated by transposon mutagenesis

More than 3000 transposon insertions were isolated and screened for loss of Sb(III) resistance with one mutant displaying lower Sb(III) resistance. This strain, TS44-gshA_540_, was selected for further characterization (data not shown). The Tn5 transposon had inserted into *gshA* encoding a putative glutamate-cysteine ligase at nucleotide 540 (Additional file 2: Figure S1A). The *gshA* encoded glutamate-cysteine ligase is the rate-limiting enzyme in *de novo* GSH biosynthesis and therefore is involved in conferring resistance to ROS and their toxic products [44]. Additionally, a strain complementing the *gshA* insertion was constructed and named TS44-gshA-C. Diagnostic PCRs were used to confirm the transposon mutation and the complementation (Additional file 2: Figure S1B-C).

### An insertion in *gshA* led to an increased Sb(III) oxidation rate

**Fig. 1:**
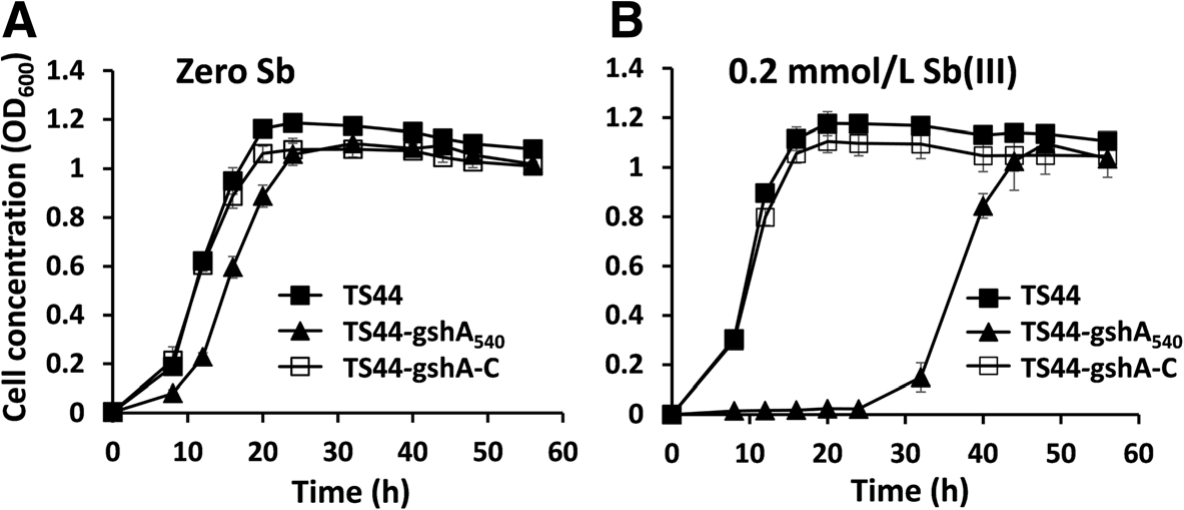
Growth curves of *P. stutzeri* strains TS44, TS44-gshA_540_ and TS44-gshA-C without (**a**) or with (**b**) the addition of 0.2 mmol/L Sb(III). Error bars correspond to the standard deviations of the means from three independent experiments

**Fig. 2:**
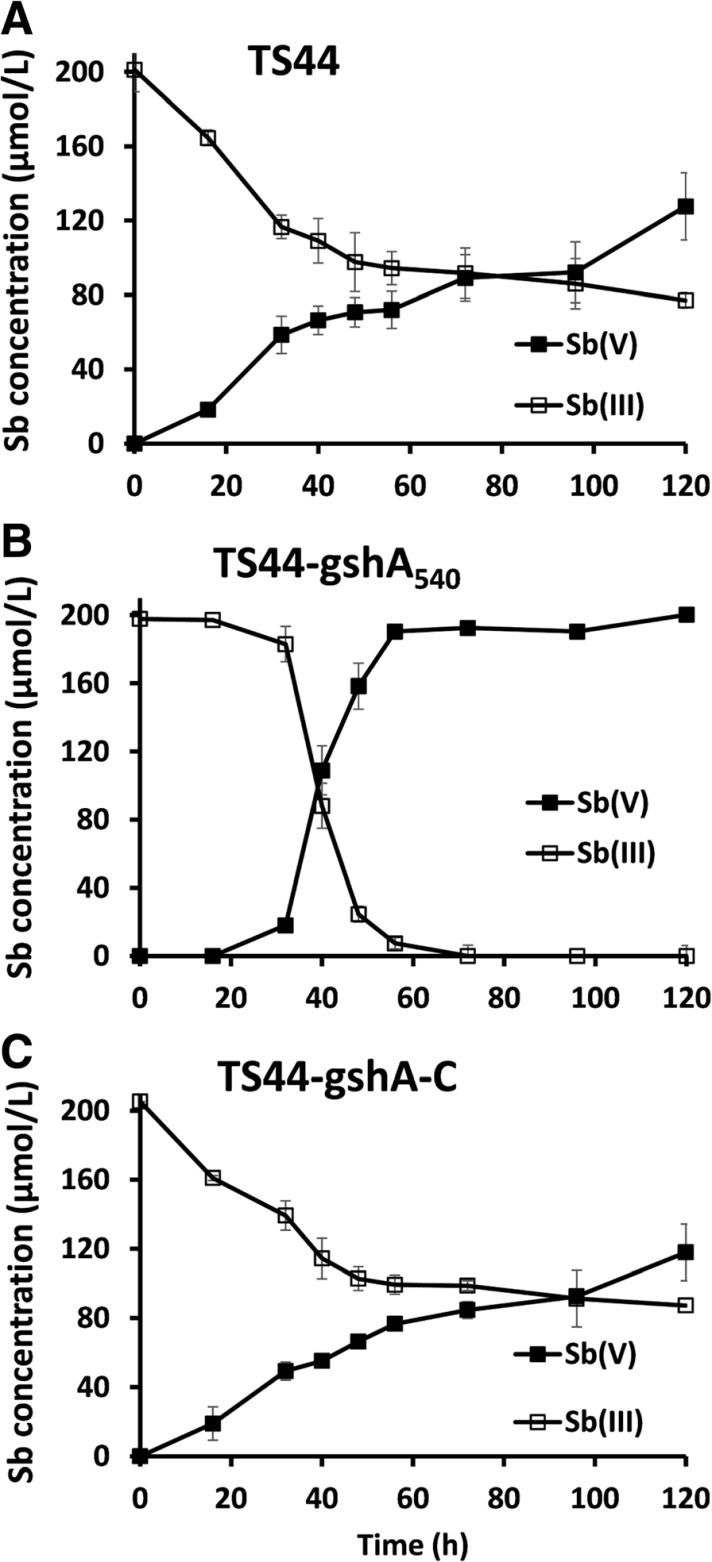
Sb(III) oxidation in *P. stutzeri* strains TS44, TS44-gshA_540_ and TS44-gshA-C with the addition of 0.2 mmol/L Sb(III). The amounts of Sb(III) and Sb(V) in culture fluids were calculated based on culture volume to normalize for the total amount of antimony added. Sb(III) and Sb(V) concentrations in the culture fluids were measured using HPLC-HG-AFS. Error bars correspond to the standard deviations of the means from three independent experiments

Cells of *P. stutzeri* TS44, TS44-gshA_540_ and TS44-gshA-C were incubated in CDM medium lacking Sb(III) supplementation after thorough washing. The growth of strain TS44-gshA_540_ was slightly slower compared to the wild type strain (Fig. 1a). The growth of strain TS44-gshA_540_ was further delayed by supplementation with 0.2 mmol/L Sb(III), causing strain TS44-gshA_540_ to need an extra 24 h to reach the lag phase (Fig. 1b). The *gshA* complementing strain TS44-gshA-C showed no difference in growth compared to the wild type strain regardless of whether Sb(III) was added or not. Although the growth of TS44-gshA_540_ was delayed, strain TS44-gshA_540_ oxidized 88% Sb(III) to Sb(V) in 48 h (Fig. 2b). In contrast, strains TS44 and TS44-gshA-C both oxidized only 48% Sb(III) to Sb(V) in 48 h (Fig. 2a and c). The Sb(III)-oxidation rate of TS44-gshA_540_ significantly increased by 83% in the monitored 48 h time frame (*p* < 0.01) (Fig. 2).

### An insertion in *gshA* eliminated GshA activity and influenced cellular contents of GSH and ROS

**Fig. 3:**
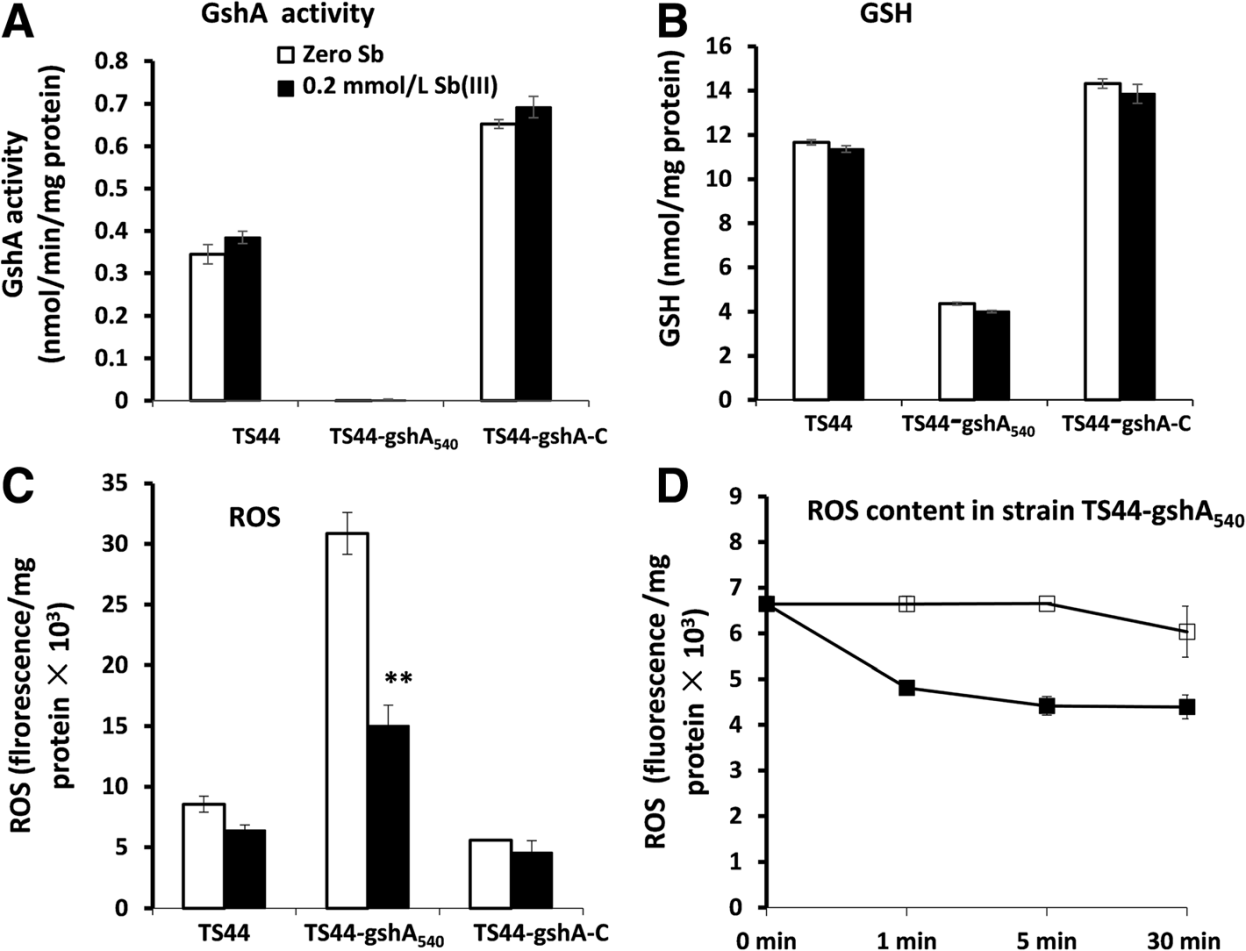
*gshA* insertion affected GSH content, GshA activity and ROS content. **a**, GshA activity, (**b**), GSH content, (**c**), ROS content and (**d**) ROS content in strain TS44-gshA_540_. Data symbols shown in panels (**a**), (**b**) and (**c**) are the same. Data are expressed as the mean ± SD, *N* = 3. **Indicates a significant difference from the control (*p* < 0.01)

We investigated both GshA activity and the GSH and ROS content to elucidate how the presence of GshA affected Sb(III) oxidation. The GshA activity in strains TS44 and TS44-gshA-C was slightly increased following the addition of Sb(III); however, GshA activity was undetectable in the insertional mutant strain TS44-gshA_540_ (Fig. 3a), indicating that the transposon insertion functionally disrupted *gshA* in strain TS44. Consistent with this result, the GSH content was decreased by approximately 66% in the mutant compared to strains TS44 and TS44-gshA-C regardless of whether Sb(III) was provided (Fig. 3b).

As a next step, cellular ROS content was determined because GSH is the central substrate for cellular protection against ROS and their toxic products [24]. The cellular ROS content in strain TS44-gshA_540_ was increased by approximately three-fold compared to strains TS44 and TS44-gshA-C regardless of whether Sb(III) was provided or not. The cellular ROS content was significantly decreased in strain TS44-gshA_540_ after 30 min of incubation with Sb(III) compared to no Sb(III) addition (Fig. 3b-c), despite the fact that *gshA* was mutated and the GSH content was obviously decreased (Fig. 3a-b). After addition of Sb(III), the ROS content changed quickly in 30 min, and decreased significantly in the strain TS44-gshA_540_ (Fig. 3d). In such a short time, the oxidation rate of Sb(III) did not significantly change. The Sb(III) oxidation mainly occurred in the 36–48 h time frame (Fig. 2).

### Sb(III) affected transcription of *gshA*, *sodB*, *sodC* and *katE* differently

The expression of *gshA*, *sodB*, *sodC* and *katE* was monitored, because GshA, SodB, SodC and KatE are all involved in changes of GSH, ROS or H_2_O_2._ There are two distinct genes (*sodB* and *sodC*) encoding superoxide dismutase (SOD) on the genome of *P. stutzeri* TS44 that encode for Fe SOD and Cu-Zn SOD, respectively. These two enzymes shared over 83% and 58% amino acid sequence similarity with the respective enzymes of other species of *Pseudomonas*, respectively, and can convert O_2_^•-^ to H_2_O_2_ and O_2_. In the *lacZ* reporter fusion analysis, we only transformed the *lacZ* fusions designated pLSP-P*gshA*, pLSP-P*sodB* and pLSP-P*sodC* into strains TS44 and TS44-gshA_540_ because the *gshA* complementary strain TS44-gshA-C carried a plasmid that might be incompatible with the *lacZ* fusion.

**Fig. 4:**
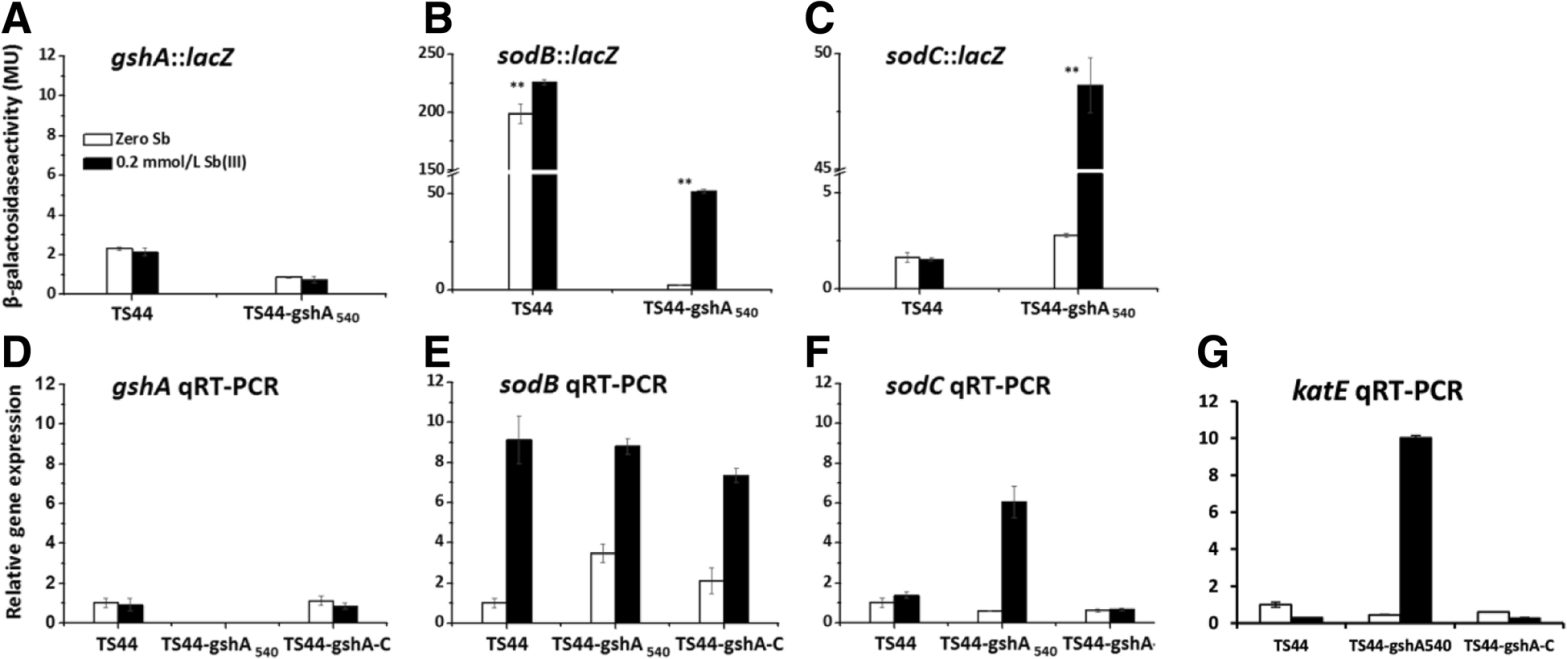
The effect of Sb(III) to the transcription of *gshA*, *sodB*, *sodC* and *katE*. The transcription of *gshA*, *sodB* and *sodC* was tested by a *lacZ* reporter fusion and qRT-PCR, while the *katE* transcription was only tested by qRT-PCR. For *lacZ* reporter fusion (**a**, **b** and **c**), data are expressed as the mean ± SD, *N* = 3. **Indicates a significant difference from the control (*p* < 0.01). For qRT-PCR, error bars correspond to the standard deviations of the means from three biological replicates. Gene expression was normalized to the 16S rRNA gene. The results are presented as the mean gene expression normalized to mRNA levels in Sb(III)-free CDM. Data symbols shown in all panels are the same

**Fig. 5:**
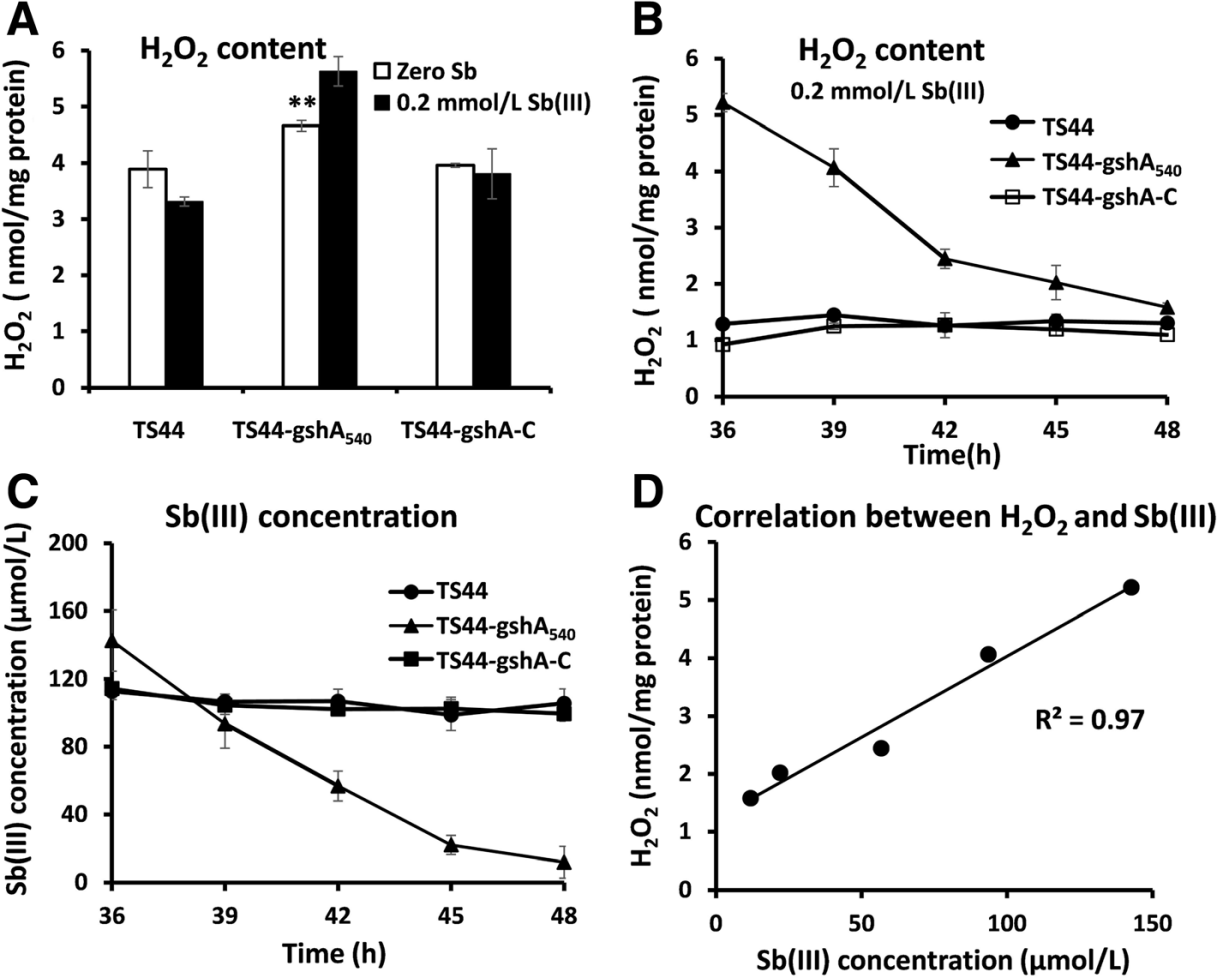
The H_2_O_2_ content is correlated with bacterial Sb(III) oxidation. The *P. stutzeri* strains were cultured as described above. **a** The H_2_O_2_ concentration was tested after 30 min of incubation with Sb(III). **Indicates a significant difference from the control (*p* < 0.01). **b** H_2_O_2_ content and (**c**) Sb(III) concentration in strains TS44, TS44-gshA_540_ and TS44-gshA-C from 36 to 48 h of incubation in cultures supplemented with 0.2 mmol/L Sb(III). Data are expressed as the mean ± SD, *N* = 3. **d** Correlation between H_2_O_2_ and Sb(III) concentrations in strain TS44-gshA_540_

The transcription levels of *gshA*, *sodB*, *sodC* and *katE* were tested by qRT-PCR in all three strains. Sb(III) had no effect on the transcription of *gshA* in strains TS44, TS44-gshA_540_ or TS44-gshA-C (Fig. 4a and d). The *gshA*::*lacZ* fusion only showed a low level of background expression levels in the *gshA* insertional mutation strain TS44-gshA_540_, (Fig. 4a), which indicated a very low promoter activity of *gshA*, while qRT-PCR analysis showed no transcription of *gshA* in strain TS44-gshA_540_ (Fig. 5d). Moreover, *lacZ* fusion and qRT-PCR both showed that the *sodB* gene was strongly induced by Sb(III) in all three strains (Fig. 4b and e). Sb(III) only induced transcription of *sodC* and *katE* in the mutant strain TS44-gshA_540_, but not in strains TS44 and TS44-gshA-C (Fig. 4c, f and g).

### Disruption of GSH synthesis allowed accumulation of H_2_O_2_

In order to examine whether the ROS-protection system is involved in H_2_O_2_ production, we tested the H_2_O_2_ content in the presence and absence of Sb(III) in all strains. The H_2_O_2_ content was slightly higher in *gshA* mutant strain TS44-gshA_540_ without addition of Sb(III) compared to strains TS44 and TS44-gshA-C (Fig. 5a). Notably, after a 30 min incubation with 0.2 mmol/L Sb(III), the H_2_O_2_ content was increased in the mutant strain TS44-gshA_540_, but slightly decreased in strains TS44 and TS44-gshA-C (Fig. 5a), indicating that the disruption of GSH synthesis is related to the accumulation of H_2_O_2_. In addition, it appears the conversion of ROS by SOD was more efficient than the H_2_O_2_ conversion by KatE and thus would both *sod* and *katE* were induced by Sb(III).

### H_2_O_2_ is responsible for Sb(III) oxidation

H_2_O_2_ was shown to be an efficient Sb(III) oxidant in vitro (Additional file 3: Figure S2) as the un-inoculated control showed no Sb(III) oxidation (Additional file 3: Figure S2). A control with dead cells after boiling the culture also showed no oxidation of Sb(III) (data not shown). As the efficiency of Sb(III) oxidation in the mutant strain TS44-gshA_540_ was shown to be highest from 32 to 48 h (Fig. 2), the H_2_O_2_ and Sb(III) content in strains TS44, TS44-gshA_540_ and TS44-gshA-C were also measured from 36 to 48 h. Interestingly, the H_2_O_2_ content in strain TS44-gshA_540_ was significantly decreased from 5.2 to 1.1 nmol/mg protein, while the H_2_O_2_ content was stable at a low level in strains TS44 and TS44-gshA-C (Fig. 5b). Correspondingly, Sb(III) was oxidized to Sb(V) concomitant with a decrease in H_2_O_2_ content, while the Sb(III) concentration was almost stable in strains TS44 and TS44-gshA-C (Fig. 5c). The consumed H_2_O_2_ content and the oxidized Sb(III) showed a linear correlation with a correlation coefficient of 0.97 (Fig. 5d), indicating that H_2_O_2_ is responsible for Sb(III) oxidation. In addition, we could show that growth was inhibited with increasing concentration of H_2_O_2_ (Additional file 4: Figure S3), thus Sb(III) oxidation may contribute to the bacterial detoxification of H_2_O_2_ and Sb(III).

## Discussion

At present, several Sb(III)-oxidizing strains have been reported [13, 14, 18]; some of these strains were also able to oxidize As(III) [13, 15, 19]. The arsenite oxidase AioAB was demonstrated to be capable of oxidizing Sb(III), but the AioAB kinetic rate of the reaction was orders of magnitude higher for As(III) than for Sb(III) [19]. Moreover, AnoA belonging to the SDR superfamily was reported to be able to catalyze Sb(III) oxidation using NADP^+^ as a cofactor [20]. However, disruption of *aioA* and *anoA* in *Agrobacterium tumefaciens* caused a decrease in Sb(III) oxidation of only about 25% [19] and 27% [20], respectively, indicating the existence of other bacterial Sb(III) oxidation mechanisms. After discovering that the presence of *gshA* affected the Sb(III) oxidation rate by transposon mutagenesis in strain TS44-gshA_540_, we proposed that some abiotic cellular components, such as GSH, ROS or H_2_O_2_, may have a role in the bacterial oxidation of Sb(III).

**Fig. 6:**
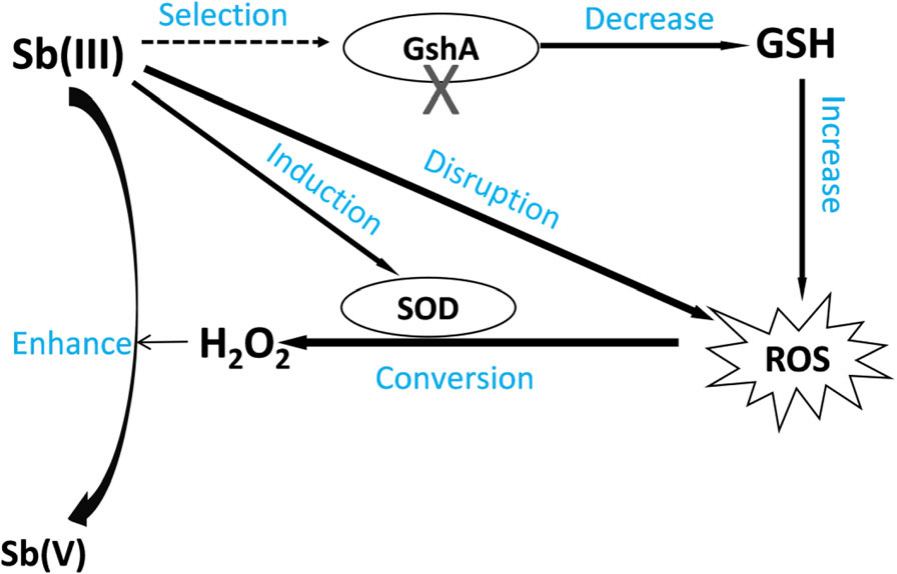
The proposed model for Sb(III) bacterial oxidation in *P. steutzeri* TS44. In this study, i) the addition of Sb(III) would trigger the ROS-protective system by inducing the transcription of *sodB*, *sodC* and *katE*, with SodB and SodC catalyzing the conversion of ROS to H_2_O_2_, while KatE responsible for the degradation of excessive H_2_O_2_ (data not shown); ii) the increased cellular H_2_O_2_ content enhanced the Sb(III) oxidation rate; and iii) the addition of Sb(III) played a selection role on the characterization of *gshA* insertion; iv) in addition, the accumulated H_2_O_2_ is partially cosumed by the upregulated catalase KatE (data not shown)

Subsequently, we conducted a comprehensive analysis. First, we found that a disruption of *gshA* caused a decrease in the cellular GSH amount; Second, it is conceivable that the *gshA* mutant strain resulted in an increase in cellular ROS content compared to the wild type; Third, we could also show a linear correlation between the decrease of H_2_O_2_ content and the increase in Sb(III) oxidation rate, indicating that Sb(III) oxidation consumed H_2_O_2_ and acts as a detoxification mechanism to counter this cellular stressor. In addition, it appeared that Sb(III) directly caused a disruption of the ROS-protection system in strain TS44-gshA_540_ and allowed the accumulation of H_2_O_2_ instead of affecting GSH levels, since neither the activity of GshA nor the GSH content were influenced by Sb(III). Previously, it was reported that Sb(III) may consume residual GSH (forming a stable Sb(GS)_3_ complex) in red blood cells [45]. However, we did not observe significant changes in GSH content when Sb(III) was added to wild type strain TS44 and the *gshA* complemented strain TS44-gshA-C, indicating little of this complex was formed and Sb(III) was mainly oxidized to Sb(V) by H_2_O_2._ We therefore propose a new model for Sb(III) oxidation in *P. stutzeri* TS44. i) the addition of Sb(III) would trigger the ROS-protective system by inducing the transcription of *sodB*, *sodC* and *katE*, with SodB and SodC catalyzing the conversion of ROS to H_2_O_2_, and KatE responsible for the depletion of excessive H_2_O_2_; ii) the increased cellular H_2_O_2_ content enhanced the Sb(III) oxidation rate; and iii) the addition of Sb(III) played a selection role on the characterization of the *gshA* insertion (Fig. 6); iv) in addition, the accumulated H_2_O_2_ was partially consumed by KatE (data not shown), since *katE* was induced by Sb(III) (Fig. 4 g), and similar result was described recently with transcription of the peroxidase-encoding gene *katA* being induced by both Sb(III) and H_2_O_2_ [46].

Several chemical substances (i.e., amorphous iron, manganese oxyhydroxides and H_2_O_2_) have been reported to be capable of mediating Sb(III) oxidation in vitro [47–49]. OH^•^ was the oxidant in acidic solutions and H_2_O_2_ was the main oxidant in neutral and alkaline solutions involved in Sb(III) oxidation, with a pH range of 8.1 to 11.7 [48, 49]. Sb(III) was reported to cause cellular oxidative stress in lymphoid tumoral cells [50]. H_2_O_2_ is a substantial component of cellular oxidative stress [51] and can inhibit cellular growth [52]. In this study, the pH of the cultures changed from the initial 7.0 to approximately 8.0 following exposure to Sb(III), indicating that H_2_O_2_ may correlate with an increase in the Sb(III) oxidation rate. In addition, the in vitro experiment provided direct evidence that H_2_O_2_ can oxidize Sb(III) to Sb(V) (Additional file 4: Figure S3) and a correlation between the concentrations of Sb(III) and H_2_O_2_ was found in vivo (Fig. 5d). As an moderatedly reactive intermediate, O_2_^•-^ may also have an effect on Sb(III) oxidation. However, in the presence of high level of SOD, the steady-state of O_2_^•-^ would be no more than 0.1 nmol/L and thus the effect of O_2_^•-^ is negligible [53]. Thus, we conclude that H_2_O_2_ oxidized Sb(III) to Sb(V).

## Conclusions

This study proposed a novel mechanism for microbial antimonite oxidation involving changes in cellular content of components related to oxidative stress (GSH, ROS and H_2_O_2_). Although H_2_O_2_ was reported to be capable of oxidizing Sb(III) chemically as early as 2005 [11], this is the first study to demonstrate that H_2_O_2_ is responsible for Sb(III)-oxidation in a microbe. The results showed that a disruption of bacterial GSH-dependent ROS-protection mechanism allowed H_2_O_2_ to build up and thus promote the oxidation of Sb(III) to Sb(V). In addition, the oxidation of Sb(III) to Sb(V) is a detoxification process against the cellular stressor H_2_O_2_. We do not exclude the possibility of an additional enzymatic process responsible for Sb(III) oxidation in strain TS44, since genes encoding a putative arsenite oxidase AioBA were found on its genome [28] which may function as a Sb(III) oxidase. Our data and other findings [19, 20, 46] could show that microbial antimonite oxidation contains both biotic and abiotic components.

## Additional files

Additional file 1: Table S1. Primers used in this study. (DOC 35 kb)
Additional file 2: Figure S1. The gene cluster containing *gshA* in strain TS44 and Diagnostic PCR confirming the transposon mutation to create mutant strain TS44-gshA_540_ and complementation to create TS44-gshA540-C. (**A**), The transposon insertion site of the *gshA* mutant is shown by the vertical arrow. (**B**), PCR used primers R6K-F/R6K-R and TnpA-F/TnpA-R (Additional file 4: Table S1) using genomic DNA of strain TS44-gshA_540_ as template. Lane 1 is the amplification of R6K fragment, while line 2 represents the TnpA fragment. (**C**), PCR used primers gshA-F/gshA-F (Additional file 4: Table S1). Lane 1, strain TS44, lane 2, *gshA* gene insertional inactivation strain TS44-gshA_540_ and lane 3, the complemented strain TS44-gshA540-C. M, the molecular weight marker (DL 2000 plus). Amplicon identities were confirmed by DNA sequencing. (TIF 104 kb)
Additional file 3: Figure S2. In vitro oxidation of Sb(III) by H_2_O_2_. (**A**) Sb(III) was added into uninoculated liquid CDM medium to a final concentration of 0.2 mmol/L. (**B**) CDM containing 0.2 mmol/L of Sb(III) and with the addition of 0, 0.02, 0.05, 0.1, 0.3, 0.4 and 0.5 mmol/L of H_2_O_2_, respectively. (TIF 59 kb)
Additional file 4: Figure S3. The tolerance of *P. stutzeri* strains TS44, TS44-gshA_540_ and TS44-gshA-C to H_2_O_2_. The strains were grown in liquid CDM medium until reaching an OD_600_ of 1.0 and then serially diluted 10-fold. The 10^0^, 10^−1^, 10^−3^, and 10^−5^ dilutions were spotted onto agar plates with different H_2_O_2_ concentrations. (TIF 426 kb)

## References

[1] Ehrlich HL (2008). Newman DK.

[2] Goyeneche-Patino DA, Valderrama L, Walker J, Saravia NG (2008). Antimony resistance and trypanothione in experimentally selected and clinical strains of *Leishmania panamensis*. Antimicrob Agents Ch.

[3] Liarte DB, Murta SM (2010). Selection and phenotype characterization of potassium antimony tartrate-resistant populations of four New World *Leishmania* species. Parasitol Res.

[4] Callahan MA. Water-related environmental fate of 129 priority pollutants. Office of Water Planning and Standards. Office of Water and Waste Management, US Environmental Protection Agency; Washington. 1979.

[5] WHO. Antimony in Drinking-water-Background. Document for Development of WHO Guidelines for Drinking Water Quality. Geneva. 2003.

[6] Filella M, Belzile N, Chen Y-W (2002). Antimony in the environment: a review focused on natural waters: I. Occurrence. Earth-Sci Rev.

[7] Filella M, Belzile N, Chen Y-W (2002). 2002b. Antimony in the environment: a review focused on natural waters: II. Relevant solution chemistry. Earth-Sci Rev.

[8] Wilson SC, Lockwood PV, Ashley PM, Tighe M (2010). The chemistry and behaviour of antimony in the soil environment with comparisons to arsenic: a critical review. Environ Pollut.

[9] Gebel T (1997). Arsenic and antimony: comparative approach on mechanistic toxicology. Chem-bio interact.

[10] De Boeck M, Kirsch-Volders M, Lison D (2003). Cobalt and antimony: genotoxicity and carcinogenicity. Mutat Res-fund Mol M.

[11] Leuz A-K, Johnson CA (2005). Oxidation of Sb III to Sb V by O_2_ and H_2_O_2_ in aqueous solutions. Geochim Cosmochim Ac.

[12] Smichowski P (2008). Antimony in the environment as a global pollutant: a review on analytical methodologies for its determination in atmospheric aerosols. Talanta.

[13] Li J, Wang Q, Zhang SZ, Qin D, Wang GJ (2013). Phylogenetic and genome analyses of antimony-oxidizing bacteria isolated from antimony mined soil. Int Biodet Biodegr.

[14] Shi Z, Cao Z, Qin D, Zhu W, Wang Q, Li M (2013). Correlation models between environmental factors and bacterial resistance to antimony and copper. PLoS One.

[15] Lehr CR, Kashyap DR, McDermott TR (2007). New insights into microbial oxidation of antimony and arsenic. Appl Environ Microb.

[16] Lialikova N (1974). *Stibiobacter senarmontii*--a new microorganism oxidizing antimony. Mikrobiologiia.

[17] Hamamura N, Fukushima K, Itai T (2013). Identification of antimony- and arsenic-oxidizing bacteria associated with antimony mine tailing. Microbes Environ/JSME.

[18] Nguyen VK, Lee J-U (2015). Antimony-oxidizing bacteria isolated from antimony-contaminated sediment–a phylogenetic study. Geomicrobiol J.

[19] Wang Q, Warelow TP, Kang Y-S, Romano C, Osborne TH, Lehr CR, et al. Arsenite oxidase also functions as an antimonite oxidase. Appl Environ Microb. 2015: AEM. 02981–14 10.1128/AEM.02981-14 PMC4345363 25576601

[20] Li JX, Wang Q, Li MS, Yang BR, Shi MM, Guo W (2015). Proteomics and genetics for identification of a bacterial antimonite oxidase in *Agrobacterium tumefaciens*. Environ Sci Technol.

[21] Halliwell B, Gutteridge JM (1999). Free radicals in biology and medicine.

[22] Bayr H (2005). Reactive oxygen species. Crit Care Med.

[23] Cabiscol E, Tamarit J, Ros J (2010). Oxidative stress in bacteria and protein damage by reactive oxygen species. Int Microbiol.

[24] Hayes JD, McLellan LI (1999). Glutathione and glutathione-dependent enzymes represent a co-ordinately regulated defence against oxidative stress. Free Radical Res.

[25] Masip L, Veeravalli K, Georgiou G (2006). The many faces of glutathione in bacteria. Antioxid Redox Sign.

[26] Janowiak BE, Griffith OW (2005). Glutathione Synthesis in *Streptococcus agalactiae* one protein accounts for γ-glutamylcysteine synthetase and glutathione synthetase activities. J Biol Chem.

[27] Cai L, Rensing C, Li X, Wang G (2009). Novel gene clusters involved in arsenite oxidation and resistance in two arsenite oxidizers: *Achromobacter* sp. SY8 and *Pseudomonas* sp. TS44. Appl Microbiol Biot.

[28] Li XY, Gong J, Hu Y, Cai L, Johnstone L, Grass G (2012). Genome sequence of the moderately halotolerant, arsenite-oxidizing bacterium *Pseudomonas stutzeri* TS44. J Bacteriol.

[29] Weeger W, Lievremont D, Perret M, Lagarde F, Hubert J-C, Leroy M (1999). Oxidation of arsenite to arsenate by a bacterium isolated from an aquatic environment. Biometals.

[30] Sambrook J, Fritsch E, Maniatis T (1989). Molecular Cloning: A Laboratory Manual.

[31] Zheng S, Su J, Wang L, Yao R, Wang D, Deng Y (2014). Selenite reduction by the obligate aerobic bacterium *Comamonas testosteroni* S44 isolated from a metal-contaminated soil. BMC Microbiol.

[32] Overbeek R, Olson R, Pusch GD, Olsen GJ, Davis JJ, Disz T (2014). The SEED and the Rapid Annotation of microbial genomes using Subsystems Technology RAST. Nucleic Acids Res.

[33] Altschul SF, Gish W, Miller W, Myers EW, Lipman DJ (1990). Basic local alignment search tool. J Mol Biol.

[34] Chen F, Cao Y, Wei S, Li Y, Li X, Wang Q, Wang G (2015). Regulation of Arsenite Oxidation by the Phosphate Two-Component System PhoBR in *Halomonas* sp. HAL1. Front Microbiol.

[35] Hanahan D (1983). Studies on transformation of *Escherichia coli* with plasmids. J Mol Biol.

[36] Simon R, Priefer U, Pühler A (1983). A broad host range mobilization system for in vivo genetic engineering: transposon mutagenesis in gram negative bacteria. Nat Biotechnol.

[37] Larsen RA, Wilson MM, Guss AM, Metcalf WW (2002). Genetic analysis of pigment biosynthesis in *Xanthobacter autotrophicus* Py2 using a new, highly efficient transposon mutagenesis system that is functional in a wide variety of bacteria. Arch Microbiol.

[38] Bradford MM (1976). A rapid and sensitive method for the quantitation of microgram quantities of protein utilizing the principle of protein-dye binding. Anal Biochem.

[39] White CC, Viernes H, Krejsa CM, Botta D, Kavanagh TJ (2003). Fluorescence-based microtiter plate assay for glutamate–cysteine ligase activity. Anal Biochem.

[40] Guo FF, Yang W, Jiang W, Geng S, Peng T, Li JL (2012). Magnetosomes eliminate intracellular reactive oxygen species in *Magnetospirillum gryphiswaldense* MSR-1. Environ Microbiol.

[41] Wang Q, Lei Y, Xu X, Wang G, Chen L-L (2012). Theoretical prediction and experimental verification of protein-coding genes in plant pathogen genome *Agrobacterium tumefaciens* strain C58. PLoS One.

[42] Miller JH. Assay of β-galactosidase. In: Experiments in Molecular Genetics. New York: Cold Spring Harbor Laboratory Press; 1972. p. 352–55.

[43] Seaver LC, Imlay JA (2001). Alkyl hydroperoxide reductase is the primary scavenger of endogenous hydrogen peroxide in *Escherichia coli*. J Bacteriol.

[44] Murata K, Kimura A (1982). Cloning of a gene responsible for the biosynthesis of glutathione in *Escherichia coli* B. Appl Environ Microb.

[45] Sun H, Yan SC, Cheng WS (2000). Interaction of antimony tartrate with the tripeptide glutathione. Eur J Biochem.

[46] Li J, Wang Q, Oremland R S, Kulp T R, Rensing C, Wang G. Microbial antimony biogeochemistry-enzymes, regulation and related metabolic pathways. Appl Environ Microb. 2016: AEM. 01375–16. 10.1128/AEM.01375-16 PMC5007761 27342551

[47] Belzile N, Chen Y-W, Wang Z (2001). Oxidation of antimony III by amorphous iron and manganese oxyhydroxides. Chem Geol.

[48] Leuz A-K, Hug SJ, Wehrli B, Johnson CA (2006). Iron-mediated oxidation of antimony III by oxygen and hydrogen peroxide compared to arsenic III oxidation. Environ Sci Technol.

[49] Kong L, Hu X, He M (2015). Mechanisms of Sb III oxidation by pyrite-induced hydroxyl radicals and hydrogen peroxide. Environ Sci Technol.

[50] Lecureur V, Le Thiec A, Le Meur A, Amiot L, Drenou B, Bernard M (2002). Potassium antimonyl tartrate induces caspase‐and reactive oxygen species‐dependent apoptosis in lymphoid tumoral cells. Brit J haematol.

[51] Imlay JA, Chin SM, Linn S (1988). Toxic DNA damage by hydrogen peroxide through the Fenton reaction in vivo and in vitro. Science.

[52] McLeod JW, Gordon J (1922). Production of hydrogen peroxide by bacteria. Biochem J.

[53] Imlay JA, Fridovich I (1991). Assay of metabolic superoxide production in *Escherichia coli*. J Biol Chem.

