# Supplementary material for: Disrupting ROS-protection mechanism allows hydrogen peroxide to accumulate and oxidize Sb(III) to Sb(V) in Pseudomonas stutzeri TS44
Source: BMC Microbiol. 2016 Nov 25;16:279. doi: 10.1186/s12866-016-0902-5 (PMC5123405; doi:10.1186/s12866-016-0902-5)
Supplement: Additional file 1: Table S1. — Primers used in this study. (DOC 35 kb) [file 12866_2016_902_MOESM1_ESM.doc]

**Table S1.** Primers used in this study

| **Primer pairs** | **Primer sequences** | **Usage** |
| --- | --- | --- |
| pRLSR/pRLSF | 5' AACAAGCCAGGGATGTAACG 3'  5' CAGCAACACCTTCTTCACGA 3' | Transposon insertion mutants sequencing |
| R6K-F/R6K-R | 5' GGCTTCTCAGTGCGTTAC 3'  5' GAGGATCTGAAGATCAGC 3' | Transposon insertion mutants verification |
| TnpA-F/TnpA-R | 5' GGCAAGAAAGCCATCCAG 3'  5' CTTTGAGTTATCGCCACC 3' | Transposon insertion mutants verification |
| gshA-F/gshA-R | 5'AAAAAGCTTCGTGCACCAGGGCGACAAG 3'  5' AAAGGATCCCGGCGAGTTGGCCTGTCT 3' | For construction of *gshA* complemented |
| RT-16S-F/RT-16S-R | 5' TCCAAAACTGGCGAGCTAGAG 3'  5' CAACGGCTAGTCGACATCGTT 3' | For RT-PCR of 16S rRNA gene |
| RT-gshA-F/RT-gshA-R | 5' CGGACGAGGCGGACATT 3'  5' CCCAAGGCAGGTAGAGC 3' | For RT-PCR of *gshA* |
| RT-sodB-F/RT-sodB-R | 5' GCCTCCTTCGGCTCGTT 3'  5' CGTCGGACTTCTTCACCAG 3' | For RT-PCR of *sodB* |
| RT-sodC-F/RT-sodC-R | 5' GACCCGAAGAATACTG 3'  5' CTTGCCATCCTTGTC 3' | For RT-PCR of *sodC* |
| RT-katE-F/RT-katE-R | 5'AAGTTCTATACCGAGCAG 3'  5'AAATCCCAGTTGTTGTTC 3' | For RT-PCR of *katE* |
| PgshA-f/PgshA-r | 5' aaagaattcTGCTGCTCATGGGAACT 3'  5' aaaggatccGCTACCTGGGTCAGTCG 3' | For construction of  P*gshA*::*lacZ* |
| Psodb-f/ Psodb-r | 5' aaagaattcGCTGACCTGGCGACTGAT 3'  5' aaaggatccGCTCGTTTCGGCTGTTCT 3' | For construction of  P*sodB*::*lacZ* |
| PsodC-f/ PsodC-r | 5' aaagaattcGAACACCGCGAAATCCA 3'  5' aaaggatccCGCCTGCTTCAGTTGAGT 3' | For construction of  P*sodC*::*lacZ* |

* The underlined sequences denote the restriction enzyme sites.
