# Supplementary figures and images for: Disrupting ROS-protection mechanism allows hydrogen peroxide to accumulate and oxidize Sb(III) to Sb(V) in Pseudomonas stutzeri TS44
Source: BMC Microbiol. 2016 Nov 25;16:279. doi: 10.1186/s12866-016-0902-5 (PMC5123405; doi:10.1186/s12866-016-0902-5)

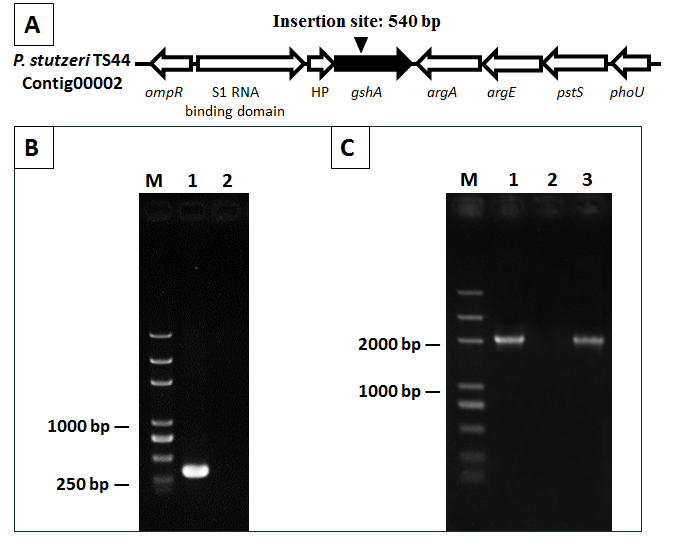

Supplement: Additional file 2: Figure S1. — The gene cluster containing gshA in strain TS44 and Diagnostic PCR confirming the transposon mutation to create mutant strain TS44-gshA540 and complementation to create TS44-gshA540-C. (A), The transposon insertion site of the gshA mutant is shown by the vertical arrow. (B), PCR used primers R6K-F/R6K-R and TnpA-F/TnpA-R (Additional file 4: Table S1) using genomic DNA of strain TS44-gshA540 as template. Lane 1 is the amplification of R6K fragment, while line 2 represents the TnpA fragment. (C), PCR used primers gshA-F/gshA-F (Additional file 4: Table S1). Lane 1, strain TS44, lane 2, gshA gene insertional inactivation strain TS44-gshA540 and lane 3, the complemented strain TS44-gshA540-C. M, the molecular weight marker (DL 2000 plus). Amplicon identities were confirmed by DNA sequencing. (TIF 104 kb) [file 12866_2016_902_MOESM2_ESM.tif]

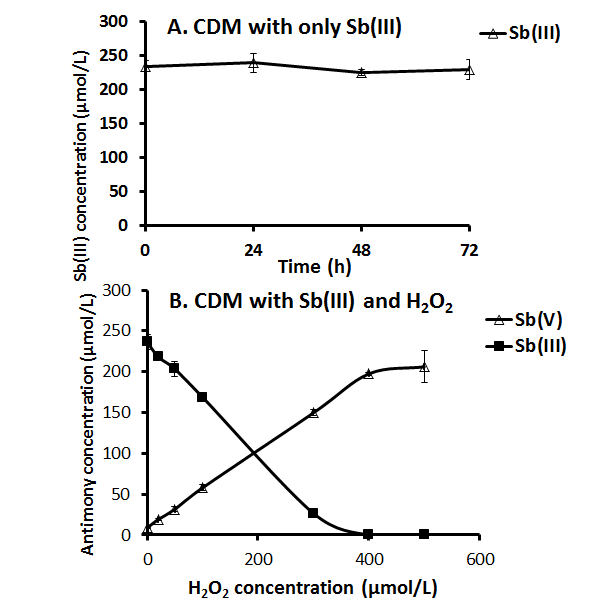

Supplement: Additional file 3: Figure S2. — In vitro oxidation of Sb(III) by H2O2. (A) Sb(III) was added into uninoculated liquid CDM medium to a final concentration of 0.2 mmol/L. (B) CDM containing 0.2 mmol/L of Sb(III) and with the addition of 0, 0.02, 0.05, 0.1, 0.3, 0.4 and 0.5 mmol/L of H2O2, respectively. (TIF 59 kb) [file 12866_2016_902_MOESM3_ESM.tif]

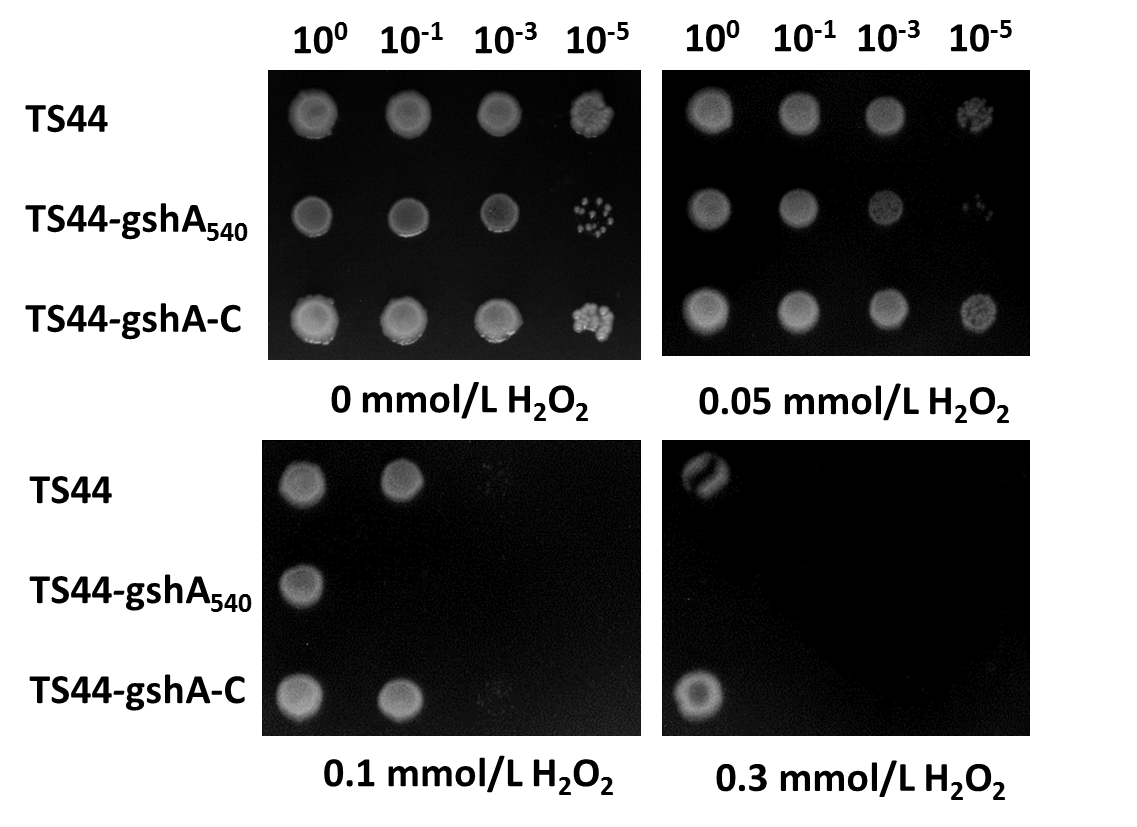

Supplement: Additional file 4: Figure S3. — The tolerance of P. stutzeri strains TS44, TS44-gshA540 and TS44-gshA-C to H2O2. The strains were grown in liquid CDM medium until reaching an OD600 of 1.0 and then serially diluted 10-fold. The 100, 10−1, 10−3, and 10−5 dilutions were spotted onto agar plates with different H2O2 concentrations. (TIF 426 kb) [file 12866_2016_902_MOESM4_ESM.tif]
